# The chances in the redox priming of nondormant recalcitrant seeds by spermidine

**DOI:** 10.1093/treephys/tpad036

**Published:** 2023-03-21

**Authors:** Hanna Fuchs, Beata P Plitta-Michalak, Arleta Małecka, Liliana Ciszewska, Łukasz Sikorski, Aleksandra M Staszak, Marcin Michalak, Ewelina Ratajczak

**Affiliations:** Institute of Dendrology, Polish Academy of Sciences, Parkowa 5, 62-035 Kórnik, Poland; Department of Chemistry, Faculty of Agriculture and Forestry, University of Warmia and Mazury in Olsztyn, Plac Łódzki 4, 10-719 Olsztyn, Poland; Institute of Molecular Biology and Biotechnology, Faculty of Biology, Adam Mickiewicz University in Poznań, Uniwersytetu Poznańskiego 6, 61-614 Poznań, Poland; Department of Epidemiology and Cancer Prevention, Greater Poland Cancer Centre, Garbary 15 Street, 61-866 Poznan, Poland; Institute of Molecular Biology and Biotechnology, Faculty of Biology, Adam Mickiewicz University in Poznań, Uniwersytetu Poznańskiego 6, 61-614 Poznań, Poland; Department of Chemistry, Faculty of Agriculture and Forestry, University of Warmia and Mazury in Olsztyn, Prawocheńskiego 17, 10-720 Olsztyn, Poland; Laboratory of Plant Physiology, Department of Plant Biology and Ecology Faculty of Biology, University of Białystok, Ciołkowskiego 1J, 15-245 Białystok, Poland; Department of Plant Physiology, Genetics and Biotechnology, Faculty of Biology and Biotechnology, University of Warmia and Mazury in Olsztyn, Oczapowskiego 1A/103, 10-719 Olsztyn, Poland; Institute of Dendrology, Polish Academy of Sciences, Parkowa 5, 62-035 Kórnik, Poland

**Keywords:** antioxidant system, DNA damage, reactive oxygen species, seed aging, seed desiccation, silver maple

## Abstract

The problems posed by seed sensitivity to desiccation and aging have motivated the development of various techniques for mitigating their detrimental effects. The redox priming of seeds in antioxidant solution to improve their postharvest performance is one of the approaches. Spermidine (Spd) was tested as an invigorating solution on nondormant recalcitrant (desiccation-sensitive) seeds of the silver maple (*Acer saccharinum* L.). The treatment resulted in an 8–10% increase in germination capacity in seeds subjected to mild and severe desiccation, while in aged seeds stored for 6 months, no significant change was observed. The cellular redox milieu, genetic stability, mitochondrial structure and function were investigated to provide information about the cellular targets of Spd activity. Spermidine improved the antioxidative capacity, especially the activity of catalase, and cellular membrane stability, protected genome integrity from oxidative damage and increased the efficiency of mitochondria. However, it also elicited a hydrogen peroxide burst. Therefore, it seems that redox priming in nondormant seeds that are highly sensitive to desiccation, although it positively affected desiccated seed performance, may not be a simple solution to reinvigorate stored seeds with a low-efficiency antioxidant system.

## Introduction

One of the key properties of seeds is their sensitivity to desiccation. Seed desiccation tolerance (DT) is considered a key evolutionary step that allowed the first plant to colonize the terrestrial environment (Berjak and Pammenter 2013, Marques et al. 2019, Farrant et al. 2020). The seeds of woody plants differ in their sensitivity to water loss. Consequently, the identification of their postharvest response to desiccation and providing optimal thermal storage conditions is crucial to prolong their life expectancy because their moisture content (MC) is usually too high to store them without predrying (Suszka et al. 1996). One of the most popular seed categorizations divides them into three categories based on their desiccation sensitivity: orthodox, recalcitrant and intermediate (reviewed by Berjak and Pammenter 2013, Pammenter et al. 2014, Kijak and Ratajczak 2020).

The reduction of ATP demand by reducing metabolic activity and respiratory processes is one of the key features in obtaining DT (Leprince et al. 1999, Michalak et al. 2021). An uncoordinated slowdown of the metabolic machinery during desiccation leads to lipid oxidation, membrane and nucleic acid damage and, consequently, to the death of desiccation-sensitive (DS) seeds, which is associated with the accumulation of reactive oxygen species (ROS; Leprince et al. 1999, Pukacka and Ratajczak 2006, Jeevan Kumar et al. 2015, Ratajczak et al. 2015, Kalemba and Ratajczak 2018, Kurek et al. 2019). Continually generated ROS in plant cells, such as the superoxide radical (O_2_∙^−^), hydrogen peroxide (H_2_O_2_), hydroxyl radical (∙OH) and singlet oxygen (^1^O_2_), are the products of oxidation–reduction (redox) reactions or activated derivatives of O_2_ (Singh et al. 2016). In photosynthetic tissues (leaves), chloroplasts are the major sources of ROS generated in plants, while in nonphotosynthetic tissues (i.e. roots, meristems or seeds), mitochondria are the largest sources of ROS (Dunn et al. 2015, Ballesteros et al. 2020, Małecka et al. 2021). Reactive oxygen species levels are determined by a tightly controlled balance between production and breakdown that is achieved by highly complex antioxidant systems. These systems possess highly efficient enzymatic and nonenzymatic antioxidant defense components to control the cascades of uncontrolled oxidation (Gill and Tuteja 2010, Dietz 2014, Noctor and Foyer 2016). The disturbance of the equilibrium between ROS and the antioxidant defense system creates a condition of oxidative stress. Although early research was focused on the toxic nature of ROS, interest has shifted over the last decade toward their emerging role as signaling molecules in a broad range of physiological processes, such as growth and development, seed germination, programmed cell death, root growth and gravitropism (Baxter et al. 2014). Significantly, ROS levels increase after seed imbibition and act as a positive signal for germination. However, above certain thresholds, ROS are either too low to allow germination or too high and affect embryo viability and therefore prevent or delay germination (Bailly et al. 2008). Thus, ROS homeostasis during germination needs to be tightly controlled, which creates an ‘oxidative window’ for germination that restricts proficient seedling development within certain borders of increased ROS levels (Bailly et al. 2008, Mhamdi and Van Breusegem 2018, Bailly 2019).

Polyamines (PAs), including putrescine (Put), spermidine (Spd) and spermine (Spm), are small aliphatic polycationic nitrogenous compounds present in almost all living organisms that function as important modulators (Hu et al. 2020). In plants, they play a complex role in the plant modulation of oxidative stress. Polyamines are considered important factors involved in the regulation of plant growth and development, such as flower, leaf and root differentiation; flower and fruit development; senescence; and the germination of seeds and pollen (Huang et al. 2017). They can increase the activities of various antioxidant enzymes in plants and therefore effectively regulate oxidative stress caused by various environmental factors. Elevated levels of endogenous Spd as well as the exogenous application of Spd induce tolerance to various abiotic stresses (Seifi and Shelp 2019). On the other hand, PAs are a source of ROS; thus, they may be considered regulators of redox homeostasis that play a dual role in plant oxidative stress (Minocha et al. 2014, Seifi and Shelp 2019).

**Figure 1:**
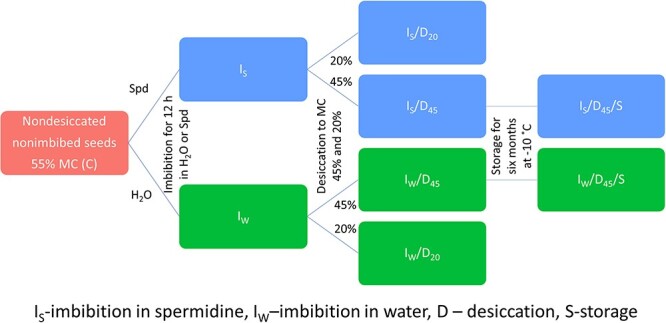
Schematic explanation of the treatments used in the research. *C*—control nondesiccated nonimbibed seeds at 55% MC; *I*_S_—seeds at 55% MC imbibed in spermidine; *I*_W_—seeds at 55% MC imbibed in water; *I*_S_/*D*_45_—seeds imbibed in spermidine desiccated to 45% MC; *I*_S_/*D*_20_—seeds imbibed in spermidine desiccated to 20% MC; *I*_W_/*D*_45_—seeds imbibed in water desiccated to 45% MC; *I*_W_/*D*_20_—seeds imbibed in water desiccated to 20% MC; *I*_S_/*D*_45_/*S*—seeds imbibed in spermidine desiccated to 45% MC stored for 6 months; *I*_W_/*D*_45_/*S*—seeds imbibed in water desiccated to 45% MC stored for 6 months.

The seed priming technique is used to improve the overall postharvest performance of seeds. In the primed state, hydration-induced specific metabolic changes affecting major cellular functions, such as protein, nucleic acid and DNA repair; ATP production; phospholipid and sterol accumulation; and the antioxidant system, are activated (Adetunji et al. 2021). Redox priming is one of the classical methods of seed pretreatment and is based on seed invigoration with antioxidant compounds, such as glutathione, tocopherol, ascorbic acid and amino acids (Adetunji et al. 2021). Interestingly, PAs have already been suggested to initiate plant defense systems and improve plant adaptability to various stresses by coping with ROS accumulation (Hu et al. 2020). It is known that PAs, including Spd, contribute significantly to the regulation of plant growth, development and metabolism. They are the regulators of many processes, such as embryogenesis, seed germination, root development, flowering, fruit development and ripening, and aging (Alcázar et al. 2010, Moschou et al. 2012, Tiburcio et al. 2014). The impact of priming with PA, particularly Spd, has already attracted interest; however, in previous research, mostly dried-stored orthodox (DT) seeds were investigated (Huang et al. 2017, Fu et al. 2019, Hu et al. 2020, Hongna et al. 2021). Importantly, orthodox and recalcitrant seeds differ significantly in the maintenance of redox balance, and recalcitrant seeds are more susceptible to damage resulting from ROS overproduction and accumulation as their protective mechanism is less efficient (Berjak and Pammenter 2013, Ratajczak et al. 2019a). Therefore, in the current research, recalcitrant nondormant seeds characterized by active metabolism and promptness to germinate after being shed were used. As Spd was described to modulate the cellular redox state (Hu et al. 2020), the aim of the current research was to expand the knowledge about the impact of PA on seeds that do not tolerate desiccation. The effect of Spd was analyzed after the treatment of seeds in several regimes: (i) imbibition, (ii) imbibition followed by desiccation to 45 and 20% MC (*I*/*D*_45_ or *D*_20_) and (iii) imbibition followed by desiccation to 45% MC and storage for 6 months (*I*/*D*_45_/*S*) (Figure 1). The specific goals were to analyze (i) the cellular redox milieu and cellular membrane state, (ii) genome integrity and (iii) the effect of Spd on the respiratory process, mitochondrial redox state and membrane integrity.

To test the effect of Spd on nondormant recalcitrant seeds, the samaras of silver maple (*Acer saccharinum* L.) were collected. Silver maple is a deciduous tree that occurs natively in eastern and central North America but is also present in Europe after being introduced in the 18th century (Burns and Honkala 1990, Suszka et al. 1996). Trees of this species prefer sunny and/or semi-shaded places and grow in river valleys and floodplains; however, they are also very often used as ornamental trees due to their early rapid growth and appealing appearance (Burns and Honkala 1990). Silver maple seeds are sensitive to water loss below 30% MC (Tylkowski 1985, Marshall et al. 2000). They can germinate in favorable conditions (temperature below 20 °C and moist soil) as soon as they fall off the trees, omitting the dormancy state (Farnsworth 2000). If the conditions are not appropriate for germination, the seeds quickly lose their viability (Pukacka et al. 2011). The humidity of freshly harvested seeds is 50–60%, and while maintaining this moisture level, they can only be stored in tightly closed containers at −3 °C (Suszka et al. 1996) for no more than 6 months (Ratajczak and Pukacka 2006).

## Materials and methods

### Preparation of research material

The seeds of the silver maple were collected in Poznań, Poland (52.43, 16.90). The seed MC (four replications) was determined by drying seeds at 103 ± 2 °C for 17 h each time the MC was analyzed. Nontreated seeds removed from the samaras were characterized by an MC of 53.4%. Seeds were soaked for 12 h in distilled water to a final MC of 57.1% or in 2.5 mM Spd solution (MC of 54.3%). After imbibition, seeds were drained and left under controlled conditions of 22 °C and 35% of relative humidity to dry to two MC levels: 45 and 20%. Next, seeds mildly desiccated to 45% MC were placed in sealed bags in a chamber at −10 °C and stored under these conditions for 6 months (Figure 1). All measurements of MC were performed prior to the imbibition stage of germination.

### Biogenic amine assay

Biogenic amines were extracted from seeds with cold 5% (v/v) hydrochloric acid (HCl) (Bouchereau et al. 2000). The extracted plant material was shaken for 1 h and centrifuged at 16,000 × *g* for 30 min at a temperature of 4 °C. The supernatants were filtered through a 0.22-μm pore nylon membrane syringe filter (Filter-Bio, Nantong City, Jiangsu, China) and stored at −20 °C. The filtrate was analyzed by ion-exchange chromatography in the AAA400 amino acid analyzer (Ingos). Biogenic amines were separated at 76 °C on a 70 × 3.7 mm column filled with POLY 8 (Ingos, Prague, Czech Republic) and were eluted from the ion-exchange column with two sodium citrate buffers (pH 5.65) with the addition of 1.0 and 2.6 M sodium chloride. The quality and quantity of the biogenic amines were assayed by postcolumn ninhydrin derivatization and photometric detection (*λ* = 570 nm). The biogenic amine standards for the chromatographic analysis were supplied by Sigma–Aldrich (St Louis, MO, USA). The quantity of biogenic amines was expressed as the mean ± SD for three replicates in each treatment.

### Germination test as a method of seed viability assessment

The germination test was conducted on five biological replicates consisting of 50 seeds in each replicate. Seeds were placed in separate containers on paper moistened in water and kept in the dark at room temperature (22 °C). Germination control was carried out daily by counting and removing germinated seeds. Seeds were considered to be germinated when the root stock reached a length of 1 cm.

### Electrolyte leakage

Seeds (four replications of samples of 10 seeds) were placed in 10 ml of deionized water. The conductivity of the solutions was measured after 24 h of incubation at room temperature using a conductivity meter InLAB 730 (Mettler Toledo, Columbus, OH, USA). The results are expressed as μS g^−1^ of dry weight (DW) (Pukacka and Wójkiewicz 2003).

### Determining the level of total H_2_O_2_ in seeds

Ten seeds per sample in four replications incubated in water or Spd were analyzed. Seeds were homogenized in 5% trichloroacetic acid (TCA). The homogenate was then centrifuged for 20 min at 4 °C at 2600 × *g*. The method of Sagisaka (1976) modified according to Ratajczak et al. (2015) was used to determine the H_2_O_2_ level. The reaction mixture contained 0.5 ml of extract, 1.5 ml of 50% TCA, 0.4 ml of 10 mM ferric ammonium sulfate and 0.2 ml of 2.5 M potassium thiocyanate. The control contained 0.5 ml of 5% TCA instead of the extract. Absorbance was measured spectrophotometrically using Shimadzu UV-2501PC spectrophotometer (Shimadzu Corporation, Kyoto, Japan) at 480 nm. The amount of H_2_O_2_ in micrograms was read from the previously prepared standard curve.

### Measurement of the antioxidant enzyme activity in seeds

Ten seeds in four replications were homogenized in liquid nitrogen, followed by the addition of an extraction buffer composed of 50-mM K-phosphate buffer containing 0.2-mM ethylenediaminetetraacetic acid (EDTA) with the addition of polyvinylpyrrolidone polymer (PVPP) (pH 7.0). The samples were then incubated on ice for 1 h, after which the extract was centrifuged for 20 min at 2600 × *g* at 4 °C. The supernatant was used to determine the activity of antioxidant enzymes.

### Determination of the catalase activity in seeds

The determination of the catalase (CAT) activity was performed using the Chance and Maehly (1955) method modified according to Ratajczak et al. (2015). The sample included 100 μl of enzyme extract, 1 ml of 30 mM H_2_O_2_ and 1 ml of 0.1 M K-phosphate buffer pH 7.0. The control contained only 2 ml of phosphate buffer. The absorbance decrease was measured every 20 s at a wavelength of 240 nm. The CAT activity was expressed as mM H_2_O_2_ mg protein^−1^ min^−1^.

### Determination of the guaiacol peroxidase activity in seeds

The guaiacol peroxidase (GPx) activity was measured spectrophotometrically based on the method of (Chance and Maehly 1955). The tests included 100 μl of protein extract, 1 ml of 0.1 M K-phosphate buffer pH 7.0, 1 ml of 1% guaiacol and 1 ml of 0.2 M H_2_O_2_. The control sample did not contain the enzyme extract. Reactions were initiated by the addition of H_2_O_2_. The absorbance was measured at a wavelength of 460 nm. The enzyme activity was calculated using the extinction coefficient *ε* = 26.6 mM cm^−1^.

### Determination of the glutathione reductase activity in seeds

The glutathione reductase (GR) activity was measured using the Foyer and Halliwell (1976) method. This enzyme catalyzes the reduction reaction of the oxidized form of glutathione (GSSG) to reduced glutathione (GSH) with the simultaneous oxidation of nicotinamide adenine dinucleotide (NADPH). The sample included 500 μl of K-phosphate buffer pH 7.5, 100 μl of enzyme extract, 100 μl of GSH, 100 μl of NaCl, 100 μl of EDTA, 100 μl of H_2_O_2_, 100 μl of NADPH and 10 μl of GR. The control sample included 500 μl of K-phosphate buffer pH 7.5, 100 μl of GSH, 100 μl of NaCl and 100 μl of EDTA. The reaction was initiated by the addition of GR. NADPH oxidation was observed as a decrease in the absorption measured spectrophotometrically every 20 s for 1 min at 340 nm.

### Measurement of the protein concentration in seeds

The protein content in the enzyme extract was determined by adding up to 50 μl of 1 ml of extract diluted 1:4 in the Bradford reagent (Bradford 1976). The absorbance was measured after 5 min on a Specord UV VIS spectrophotometer (Analytik Jena, Jena, Germany) at 595 nm. From the standard curve for bovine serum albumin (BSA) (*R*^2^ = 0.9997), the protein concentration was determined.

### Isolation of mitochondria

The seeds (10 seeds per sample in four replications) were homogenized in the isolation buffer containing 5% BSA, 1 mM EDTA, 1% PVP, 0.35 M sucrose and 0.05 M K-phosphate buffer (pH 7.2). The homogenate was centrifuged for 10 min at 3000 × *g*. Then, the supernatant was centrifuged for 20 min at 10,000 × *g*. The pellet was gently resuspended in a solution containing 0.3 M mannitol, 0.2% BSA, 1 mM EDTA and 20 mM 3-(N-morpholino)propanesulfonic acid (MOPS) (pH 7.2) and then purified in a continuous gradient formed by 24% (v/v) Percoll in 0.25 M sucrose, 0.2% BSA and 20 mM MOPS (pH 7.2). The gradient was centrifuged at 40,000 × *g* for 30 min. Afterward, the mitochondrial fractions were carefully collected, washed to remove Percoll in a 20-fold volume of buffer (0.35 M sucrose, 20 mM MOPS, pH 7.2) and centrifuged for 20 min at 4000 × *g*. The purified mitochondria were resuspended in 0.05 M K-phosphate (pH 7.5) with 0.35 M sucrose.

### Determination of changes in the structure of mitochondria using a confocal microscope

The isolated mitochondria were suspended in 0.05 M K-phosphate buffer pH 7.5 containing 0.35 M sucrose, stained with 2.5 M rhodamine 123 and left in the dark for 40 min. After incubation, mitochondria were washed two to three times with a K-phosphate buffer and imaged using a confocal microscope (Zeiss LSM 510, Axioverd 200 M, Jena, Germany) equipped with a filter set no. 10, with excitation at 450–490 nm and emission at 520 nm or more.

### Determination of the level of H_2_O_2_ in isolated mitochondria

The level of H_2_O_2_ in mitochondria isolated from seeds was determined according to Patterson et al. (1984). The decrease in absorbance was measured at 508 nm. The reaction mixture contained 50 mM K-phosphate buffer pH 8.4, 0.6 mM 4-(−2 pyridylazo)resorcinol and 0.6 mM potassium-titanium oxalate. The amount of H_2_O_2_ was calculated based on the standard curve (*R*^2^ = 0.9968) for a 0.5–25 μM concentration range and presented in nmol H_2_O_2_ mg^−1^ of protein estimated in the isolated subcellular fractions.

### Determination the level of malondialdehyde in isolated mitochondria

The malondialdehyde (MDA) content was determined by reaction with thiobarbituric acid (TBA) (Heath and Packer 1968). Mitochondria from seeds were submerged in 20% TCA with 0.5% TBA and incubated at 80 °C for 20 min. Thereafter, the solution was immediately cooled on ice to stop the reaction and centrifuged at 10,000 × *g* for 5 min. The absorbance at 532 and 600 nm was determined, and the MDA concentration was estimated by subtracting the nonspecific absorption at 600 nm from the absorption at 532 nm using an extinction coefficient *ε* = 156 mM^−1^ cm^−1^.

### Determination of CAT activity in isolated mitochondria

The activity of CAT in mitochondria was determined by direct measurement of H_2_O_2_ decomposition at 240 nm for 3 min as described by Aebi (1974) in 50 mM K-phosphate buffer (pH 7.0) containing 5 mM H_2_O_2_ and enzyme extract. The CAT activity was determined using the extinction coefficient *ε* = 36 mM^−1^ cm^−1^ for H_2_O_2_.

### Single-cell gel electrophoresis (the comet assay)

The comet assay was conducted using a previously published protocol (Plitta-Michalak et al. 2022*b*). Embryonic axes were excised from seeds and chopped with a fresh razor blade in 50-mM Sörensen buffer pH 6.8 with 0.5-mM disodium ethylenediaminetetraacetate (Na_2_EDTA) and 0.5% dimethyl sulfoxide (DMSO). Three biological replicates comprised of five axes were utilized in the assay of each treatment condition. The suspension was subsequently transferred to 1.5-ml Eppendorf tubes and kept at 4 °C for another 10 min to separate the nuclei from the cellular debris. The nuclear suspension (100 μl) was then gently mixed with 1% low-melting-point agarose (LMP agarose; TopVison Low Melting Point Agarose; Thermo Fisher Scientific, Waltham, MA, USA) prewarmed to 42 °C. Subsequently, 50 μl aliquots were placed on three microscope slides precoated with 1% normal melting-point agarose for (i) the alkaline comet assay to measure single- and double-strand breaks (SBs) and alkali-labile sites; (ii) the enzyme-modified version to measure 8-oxo-7,8-dihydroguanine (8-oxoG; + FPG; formamidopyrimidine DNA glycosylase); or (iii) the buffer control for the enzyme-modified version (−FPG). The slides were left in a lysis buffer (2.5 M NaCl, 100 mM Na_2_EDTA, 1% Triton X-100, 10 mM tris(hydroxymethyl)aminomethane (Tris) and 10% DMSO pH 10) for 1 h at 4 °C to remove histones, disrupt nucleosomes and the nuclear membrane and prepare the resulting nucleoids. Slides for the enzyme-modified comet assay (±FPG) were washed three times for 5 min with an FPG buffer (50 mM Tris–HCL, 2 mM Na_2_EDTA, 50 mM KCl, pH 7.5). Next, 50 μl of FPG enzyme (0.6 U ml^−1^) in an FPG reaction buffer supplemented with BSA (0.2 mg ml^−1^) was added to the gel, and the slides were covered with parafilm M. Only the FPG reaction buffer was added to the control slides (−FPG) to allow the determination of net incisions. The slides were incubated at 37 °C for 30 min in a humidified box. Afterward, the slides were rinsed twice with 0.4 M Tris–HCl pH 7.5 and placed in a dish containing a freshly prepared, cold, alkaline solution (1 mM Na_2_EDTA, 300 mM NaOH, pH > 13) at 4 °C for 40 min to allow DNA to unwind prior to electrophoresis in a vertical tank (20 min at 1 V cm^−1^). The electrophoretic solution was kept cold. Neutralization was performed using 0.4 M Tris–HCl pH 7.5 twice for 5 min, and then, the slides were dehydrated in methanol for 10 min and dried overnight at room temperature. The slides were stained with SybrGold (dilution: 1:10,000, Invitrogen, Waltham, MA, USA) for 1 h and washed in double-distilled H_2_O prior to imaging. Typically, 50 randomly selected comets, in the three biological replicates collectively, were scored using a fluorescence microscope (Leica TCS SP5 II, Leica Microsystems, Wetzlar, Germany), and the level of DNA SBs (% tail DNA) of the comets was recorded using Comet Assay IV analysis software (Perceptive Instruments, Haverhill, UK).

### DNA isolation and enzyme-linked immunosorbent analysis of 8-oxoG

Total genomic DNA was extracted from embryonic axes after the homogenization of the tissue in liquid nitrogen using a NucleoSpin Plant II Kit (Macherey-Nagel, Düren, Germany) according to the manufacturer’s instructions. Each experiment was replicated three times, and five embryonic axes were used in each replicate. The DNA concentration and quality (*A*_260_/*A*_280_ = 1.8–1.9) were measured with a NanoQuant Plate M200 PRO (Tecan, Männedorf, Switzerland). 8-oxoG was analyzed with an EpiQuik 8-OHdG DNA Damage Quantification Direct Kit (Epigentek, Farmingdale, NY, USA) according to the manufacturer’s instructions. Two hundred nanograms of DNA isolated from five embryonic axes was analyzed in three biological and two technical replicates as previously published (Plitta-Michalak et al. 2022*a*). The amount of 8-oxoG was read from the prepared standard curve (*R*^2^ = 0.9702).

### Oxygen consumption rate measurement in seed axes

For multiple axes respiration measurements, an XF HS Mini Analyzer (Agilent Technologies, Inc., Santa Clara, CA, USA) was used according to the method of Sew et al. (2013). Seed axes (three replicates of one ax per well) were placed in eight-well XFp sensor cartridges (Agilent Technologies) and surface-sterilized by soaking for 7 min in 12.5% (w/v) NaClO and 0.1% (v/v) Tween 20, followed by two washing steps with distilled H_2_O. After this, wells were filled with 200 μl of the respiration medium (5 mM KH_2_PO_4_, 10 mM TES, 10 mM NaCl, 2 mM MgSO_4_, pH 7.2) and loaded into the plate reader after the calibration steps using Seahorse XF Calibrant Solution (Agilent Technologies). Oxygen concentrations were determined by 75 cycles of mixing (3 min) and measurement (3 min). The oxygen consumption rate (OCR) of seed axes was analyzed using Wave v. 2.6.1 Software (Agilent Technologies).

### Statistical analysis

For statistical analyses and a graphical visualization of data, R software (R Core Team 2020) was used. The data were divided into four datasets as separate analyses were conducted to assess the effect of soaking in water and Spd on the level of biogenic amines, the effect of desiccation or storage on changes in the cytosol of silver maple seeds, and separate statistical analyses were also conducted to assess the effect of Spd treatment on the level of MDA, level of H_2_O_2_ or CAT activity in mitochondria. The one-way ANOVA was conducted to assess the effect of soaking in water and Spd on the level of biogenic amines. The effect of desiccation or storage on germination was evaluated separately using a generalized linear model (GLM) with a binomial distribution. The impact of desiccation or storage on the level of electrolyte leakage (EL); the level of H_2_O_2_; the level of MDA; DNA strand damage; the level of 8-oxoG [analyzed by the enzyme-modified comet assay and enzyme-linked immunosorbent analysis (ELISA)]; or the activity of CAT, GPx or GR was evaluated using a linear model. All models used to assess the influence of desiccation or storage on silver maple seeds included the main effects and interactions between seed MC and seed treatment. The normality of the data was assessed using the Shapiro–Wilk test. For nonnormally distributed data, the most appropriate method of transformation was applied. Box−Cox transformation was used for the GPx activity, and sqrt(×) transformation was used for the GR activity and the level of EL. The level of Spd, Spm and H_2_O_2_ both in the cytosol and mitochondria, the level of DNA damage (DNA SBs and 8-oxoG), the level of MDA in mitochondria and the activity of CAT in mitochondria were transformed by the Lambert W × F transformation. The two-way ANOVA was used to test for significant differences between the mean values. Pairwise comparisons between treatments were performed with the application of Duncan’s multiple range test at *P* ≤ 0.05. For correlation, we used Spearman’s rank correlation. Hierarchical cluster analysis (HCA) was conducted on both desiccation and storage datasets. Before conducting HCA and principal component analysis (PCA), all data were scaled. The HCA was conducted on significantly correlated variables with germination, which means that in the case of the desiccation dataset, HCA was conducted on the level of EL, MDA, H_2_O_2_ and the activity of GPx and GR. In the case of the storage dataset, HCA was conducted on the level of EL, the level of MDA, the activity of GPx, the level of DNA damage and the level of 8-oxoG measured both by the enzyme-modified comet assay and ELISA test. Euclidean distances were based on a complete dataset including all replicates.

Principal component analysis was applied to the correlations of germination; the level of EL; the level of MDA; the level of H_2_O_2_; and the activity of CAT, GPx and GR.

The R-package ‘corrplot’ was used for the analysis and construction of the correlation between measured traits. The HCA was carried out in R with the factoextra package. The R-packages ‘ggplot2’, ‘factoextra’ and ‘FactoMineR’ were used for PCA. For a graphical visualization of the data, the R-package ‘ggplot2’ was used**.**

## Results

### Biogenic amine content in silver maple seeds

The following biogenic amines were identified in the seeds of silver maple: a monoamine—histamine (His), diamines: agmatine (Agm) and Put, a triamine—Spd and a tetramine—Spm. The control, nondesiccated and nonimbibed seeds at MC 55% (*C*) contained 60.09 μg g^−1^ of fresh weight (FW) of His, 35.24 μg g^−1^ FW of Agm, 9.82 μg g^−1^ FW of Put, 58.58 μg g^−1^ FW of Spd and 33.58 μg g^−1^ FW of Spm. In seeds at MC 55% imbibed in Spd (*I*_s_), Spd content was dominant (316.35 μg g^−1^ FW) by 5.4 and 3.29 times more than in *C* seeds and in seeds at MC 55% imbibed in water (*I*_w_). The content of Agm, His, Spm and Put was unaffected in seeds treated with Spd (*I*_S_) and water (*I*_W_) (Table 1).

**Table 1:** Measurement of biogenic amine content in the seeds of silver maple. *C*—control seeds, *I*_W_—seeds imbibed in water, *I*_S_—seeds imbibed in Spd.

Treatment	Biogenic amine content (μg μg^−1^ FW)				
	His	Put	Spd	Agm	Spm
*C*	60.1 ± 2.2	9.8 ± 0.9	58.6 ± 2.5^**2**^	35.2 ± 0.4	33.6 ± 2.1
*I*_W_	55.3 ± 1.8	10.4 ± 1.4	96.0 ± 36.6^**2**^	31.0 ± 3.3	24.4 ± 7.0
*I*_S_	56.1 ± 13.6	9.2 ± 2.3	316.4 ± 39.0^**1**^	27.1 ± 1.8	22.3 ± 5.9

Values marked with different upper index numbers are significanlty different at *P* ≤ 0.05 according to Duncan’s multiple range test.

### Seed viability assessment

**Figure 2:**
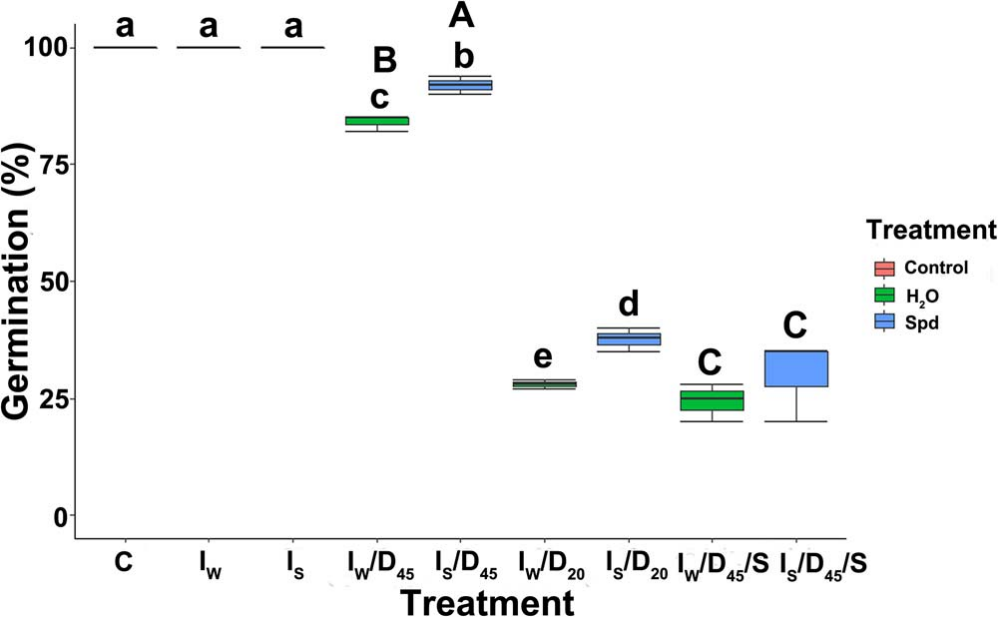
The viability of silver maple seeds based on the total germination of seeds after imbibition (*I*) and imbibition followed by desiccation (*I*/*D*) or desiccation and storage (*I*/*D*/*S*). *C*—control fresh seeds at 53% MC, *I*_S_—seeds at 55% MC imbibed in Spd, *I*_W_—seeds at 55% MC imbibed in water, *I*_S_/*D*_45_—seeds imbibed in Spd desiccated to 45% MC, *I*_W_/*D*_45_—seeds imbibed in water desiccated to 45% MC, *I*_S_/*D*_20_—seeds imbibed in Spd desiccated to 20% MC, *I*_W_/*D*_20_—seeds imbibed in water desiccated to 20% MC, *I*_S_/*D*_45_/*S*—seeds imbibed in Spd desiccated to 45% MC stored for 6 months, *I*_W_/*D*_45_/*S*—seeds imbibed in water desiccated to 45% MC stored for 6 months. Values labeled with different letters are significantly different at *P* ≤ 0.05 according to Duncan’s multiple range test. Data represent the mean ± SE (*n* = 5). Statistical analyses were conducted on separate datasets. Small letters correspond to the analysis of the effect of desiccation, and capital letters correspond to the analysis of the effect of storage.

The viability of silver maple seeds was assessed based on the total germination of control, imbibed in water or Spd (*I*), desiccated (*I*/*D*_20_ or *D*_45_) and stored seeds (*I*/*D*_45_/*S*). A significant decrease in the germination of seeds, both those treated and not treated with Spd, was observed along with the loss of water (Figure 2). When the MC of seeds prior to germination was ~55%, their germination capacity equaled 100% (Figure 2). Differences in seed germination appeared as the MC decreased. At a seed MC of 45%, the number of germinated seeds treated with Spd was 92%, which was higher than that of seeds incubated in water (84%). After reducing the MC to ~20%, the germination of seeds treated with Spd dropped to 38%, while seeds incubated in water germinated at 28% (Figure 2). After seeds at an MC of ~45% were stored for 6 months, a significant decrease in seed germination capacity was observed from 92 to 30% in the case of seeds incubated with Spd, and in the case of seeds incubated in water, germination decreased from 84 to 24% (Figure 2).

### Assessment of cell membrane conditions

**Figure 3:**
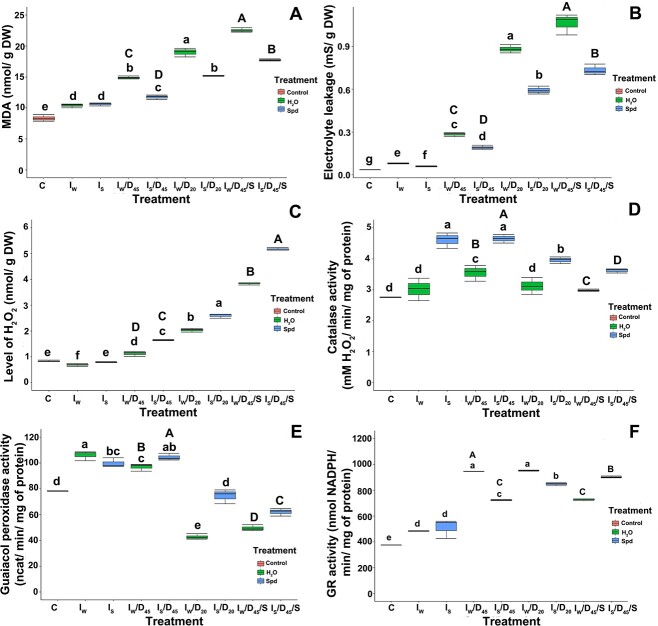
Values of (A) MDA; (B) EL; (C) H_2_O_2_ accumulation in the cytoplasm; (D) catalase activity; (E) guaiacol peroxidase activity; and (F) GR activity of silver maple seeds after imbibition (*I*) and imbibition followed by desiccation (*I*/*D*) or storage (*I*/*D*/*S*). *C*—control nondesiccated nonimbibed seeds at 53% MC, *I*_S_—seeds at 55% MC imbibed in Spd, *I*_W_—seeds at 55% MC imbibed in water, *I*_S_/*D*_45_—seeds imbibed in Spd desiccated to 45% MC, *I*_W_/*D*_45_—seeds imbibed in water desiccated to 45% MC, *I*_S_/*D*_20_—seeds imbibed in Spd desiccated to 20% MC, *I*_W_/*D*_20_—seeds imbibed in water desiccated to 20% MC, *I*_S_/*D*_45_/*S*—seeds imbibed in Spd desiccated to 45% MC stored for 6 months, *I*_W_/*D*_45_/*S*—seeds imbibed in water desiccated to 45% MC stored for 6 months. Values labeled with different letters are significantly different at *P* ≤ 0.05 according to Duncan’s multiple range test. Data represent the mean ± SE (*n* = 4). Statistical analyses were conducted on separate datasets. Small letters correspond to the analysis of the effect of desiccation, and capital letters correspond to the analysis of the effect of storage.

The MDA in nontreated control seeds was initially at a low level of 8.33 nmol g^−1^ DW. Only slightly higher levels were observed in seeds treated with Spd and water, 10.37 and 10.61 nmol g^−1^ DW, respectively (Figure 3A). With a two-step decrease in the seed MC, the level of MDA significantly increased; however, in seeds treated with Spd, it was lower than that in seeds treated with water (Figure 3A). After a storage period of 6 months, MDA levels increased significantly; nevertheless, as in the previous treatment, the MDA level was higher in seeds imbibed in water than in Spd.

Initially, only slight differences in EL were observed between untreated control seeds and seeds treated with water or Spd, and it remained in the range of 0.035–0.081 mS g^−1^ DW (Figure 3B). Along with the loss of MC, the amount of EL from seed cells increased. A difference in efflux between samples treated with water and Spd was also observed (Figure 3B). In seeds dried to 45% MC, the leakage of electrolytes was lower by ~0.1 mS g^−1^ DW in seeds treated with Spd compared with seeds treated with water. However, when the seed MC dropped to ~20%, the difference in EL between the seeds of those two treatments was close to 0.3 mS g^−1^ DW, while the EL for seeds treated with Spd was lower compared with seeds treated with water (Figure 3B). As a result of the storage of seeds, the EL increased to 0.732 mS g^−1^ DW in seeds treated with Spd and to the highest level of 1.063 mS g^−1^ DW for stored seeds followed by imbibition with water.

### The effect of Spd on the total level of H_2_O_2_

In comparison to seeds imbibed either in water or in Spd (*I*), the level of H_2_O_2_ increased in imbibed seeds subsequently desiccated (*I*/*D*) and imbibed seeds subsequently desiccated and stored (*I*/*D*/*S*). We observed an increase in the level of H_2_O_2_ in the cytosol after the desiccation of seeds to an MC of 45% in the *I*/*D* regime. Interestingly, we observed a higher amount of H_2_O_2_ in the seeds treated with Spd (1.64 nmol g^−1^ DW) than in seeds treated with water (1.12 nmol g^−1^ DW) (Figure 3C). Additionally, when seeds were desiccated to a lower MC of 20%, we observed a higher level of H_2_O_2_ (2.59 nmol g^−1^ DW) in seeds treated with Spd than in seeds treated with water (2.03 nmol g^−1^ DW) (Figure 3C). After the storage of seeds for half a year, an increase in the level of H_2_O_2_ in seeds was observed, and it was higher in seeds treated with Spd (Figure 3C).

### The effect of Spd on the activity of total antioxidant enzymes

CAT activity in untreated and water-imbibed seeds did not change at high MC (55%), although it was higher in Spd-treated seeds (Figure 3D). The enzyme activity increased in seeds mildly desiccated after imbibition (*I*/*D*_45_); however, a decrease in CAT activity was observed after desiccation to lower MC (*I*/*D*_20_). Catalase activity in stored seeds (*I*/*D*_45_/*S*) was lower than that in seeds subjected only to desiccation (*I*/*D*_45_). In the case of seeds imbibed in Spd, CAT activity was higher than that in seeds treated with water when measured after all applied regimes. However, a decrease was also observed both in seeds that were imbibed and further desiccated up to 45% (*I*/*D*_45_) and in seeds that were subsequently stored (*I*/*D*_45_/*S*) (Figure 3D).

The GPx activity was higher in seeds imbibed in water or Spd in comparison with control nontreated seeds. However, desiccation up to 45% MC of Spd-treated seeds did not affect GPx activity. Nevertheless, further desiccation to 20% (*I*/*D*_20_) and storage at 45% MC (*I*/*D*_45_/*S*) resulted in a decrease in enzyme activity; however, in seeds imbibed in Spd, the decrease was more substantial (Figure 3E).

The GR activity was higher in imbibed seeds than in the control seeds and increased after desiccation to 45% and 20% MC, but in Spd-imbibed seeds, it was lower. In stored seeds, GR was higher after Spd treatment (Figure 3F).

### The effect of Spd on DNA integrity

**Figure 4:**
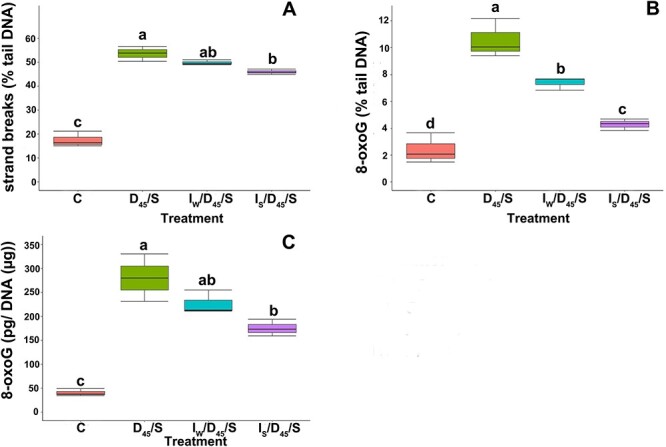
DNA integrity in embryonic axes of silver maple after the storage of control nontreated seeds and seeds imbibed in water or Spd followed by mild desiccation and storage (*I*/*D*_45_/*S*). (A) DNA SBs detected in the alkaline comet assay; (B) 8-oxoG detected in the enzyme-modified comet assay; (C) 8-oxoG detected by ELISA. *C*—control nondesiccated nonimbibed seeds at 53% MC, *D*_45_/*S*—nonimbibed seeds dried to 45% and stored for 6 months, *I*_S_/*D*_45_/*S*—seeds imbibed in Spd desiccated to 45% MC stored for 6 months, *I*_W_/*D*_45_/*S*—seeds imbibed in water desiccated to 45% MC stored for 6 months. Values labeled with different letters are significantly different at *P* ≤ 0.05 according to Duncan’s multiple range test.

The basal level of the percentage of tail DNA in nonstored nonimbibed control seeds was 17.5% (Figure 4A). The storage of control seeds for 6 months at an MC of 53.4% resulted in an increase in detected DNA SBs. When seeds were imbibed in water or Spd followed by desiccation to 45% MC and subsequently stored (*I*/*D*_45_/*S*), the relative percentage of DNA SBs was lower after treatment with Spd (45.9%). The relative amount of 8-oxoG detected in the enzyme-modified comet assay was the highest in stored control seeds, equaling 10.5% (Figure 4B). However, the treatment with water and Spd prior to storage resulted in a decrease in the relative amount of the oxidized form of guanine, with the lowest amount (4.29%) in seeds treated with Spd. The basal level of 8-oxoG was 2.40% tail DNA. Using ELISA, the treatment-related differences were confirmed, and the absolute amount of 8-oxoG was 175.4 pg μg^−1^ DNA in Spd-treated axes (*I*/*D*_45_/*S*), while in control axes, the amount of 8-oxoG was 40.6 pg μg^−1^ DNA. The highest level of 8-oxoG was observed in the axes of control seeds not imbibed prior to storage (280.1 pg μg^−1^) (Figure 4C).

### The impact of Spd on the structure of mitochondria in silver maple seeds

**Figure 5:**
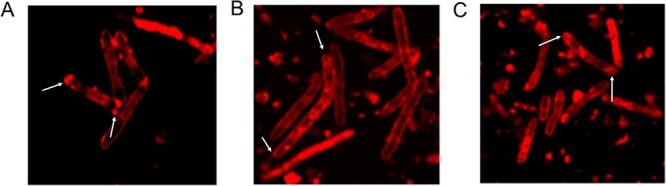
Fluorescence images of mitochondrial structure changes in silver maple seeds (A) after desiccation to 20% MC, (B) after treatment with Spd and (C) after treatment with water.

The differences in the ultrastructure of mitochondria in seeds dried to an MC of 20% and then soaked in Spd and water (control) (Figure 5) were determined using confocal microscopy. Mitochondria isolated from seeds that were dried and then treated with water or Spd have conjugated mitochondrial membranes, an oblong shape and visible mitochondrial crests. However, it was observed that the mitochondria from the seeds treated with Spd were more elongated; more cristae were visible, and they were in larger clusters. This indicates that they were very metabolically active. The seed mitochondria desiccated to 20% MC were smaller, were found in smaller clusters and had fewer visible combs, which is characteristic of cells that do not exhibit active oxygen metabolism.

### The effect of Spd and water imbibition on mitochondrial membrane conditions, the level of H_2_O_2_ and the activity of CAT in mitochondria

**Figure 6:**
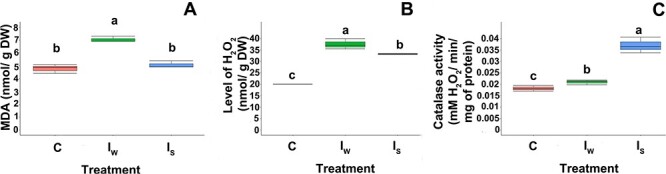
The effect of seed imbibition (*I*) in water or Spd on the amount of (A) MDA, (B) H_2_O_2_ and (C) CAT activity in silver maple seeds. *C*—control nondesiccated nonimbibed seeds at 53% MC, *I*_S_—seeds at 55% MC imbibed in Spd and *I*_W_—seeds at 55% MC imbibed in water. Seeds were imbibed for 12 h in water or 2.5 mM Spd to a final MC of ~55%. Values labeled with different letters are significantly different at *P* ≤ 0.05 according to Duncan’s multiple range test. Data represent the mean ± SE (*n* = 4).

The MDA level in the mitochondria of seeds imbibed in water was ~30% higher (7.03 nmol g^−1^ DW) than that in the mitochondria of seeds treated with Spd (4.78 nmol g^−1^ DW). There were no significant differences in MDA levels in mitochondria from Spd-treated seeds and control nonimbibed seeds (5.07 nmol g^−1^ DW) (Figure 6A).

The lowest level of H_2_O_2_ was recorded in the mitochondria of nontreated seeds at 20 nmol g^−1^ DW. The highest level of H_2_O_2_ (37.54 nmol g^−1^ DW) was observed in the mitochondria of seeds imbibed in water, and it was significantly higher in comparison with those seeds imbibed in Spd (33.22 nmol g^−1^ DW) (Figure 6B). The lowest CAT activity was observed in control nontreated seeds. The activity of CAT was induced by imbibition in water and reached the highest activity level in the variant treated with Spd. Imbibition in Spd resulted in enzyme activity twice as high as that in control seeds (Figure 6C).

### The impact of Spd on embryonic axes respiration

**Figure 7:**
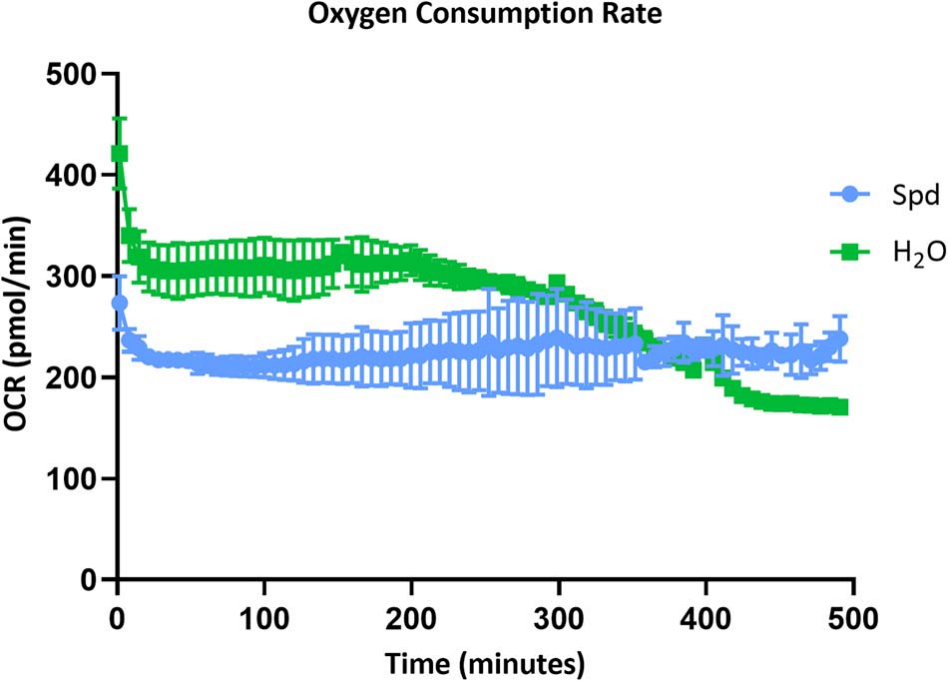
Results of the OCR measurements of seed axes imbibed in 2.5 mM Spd (blue) or water (green) for 12 h. Data represent the mean ± SE (*n* = 3).

To gain further insight into the effect of Spd on seed metabolism, we measured the OCR of axes of seeds that were treated for 12 h with 2.5 mM Spd or with water (control) for 500 min. We observed that the initial OCR was higher in the control than in the Spd-treated axes (Figure 7). Furthermore, axes treated with Spd showed stabilized OCR compared with the control group. At ~300 min of OCR measurement, the rate in the control group drastically decreased, while in the Spd-treated group, the OCR remained stable.

### Statistical analyses: correlation coefficient, HCA and PCA

**Figure 8:**
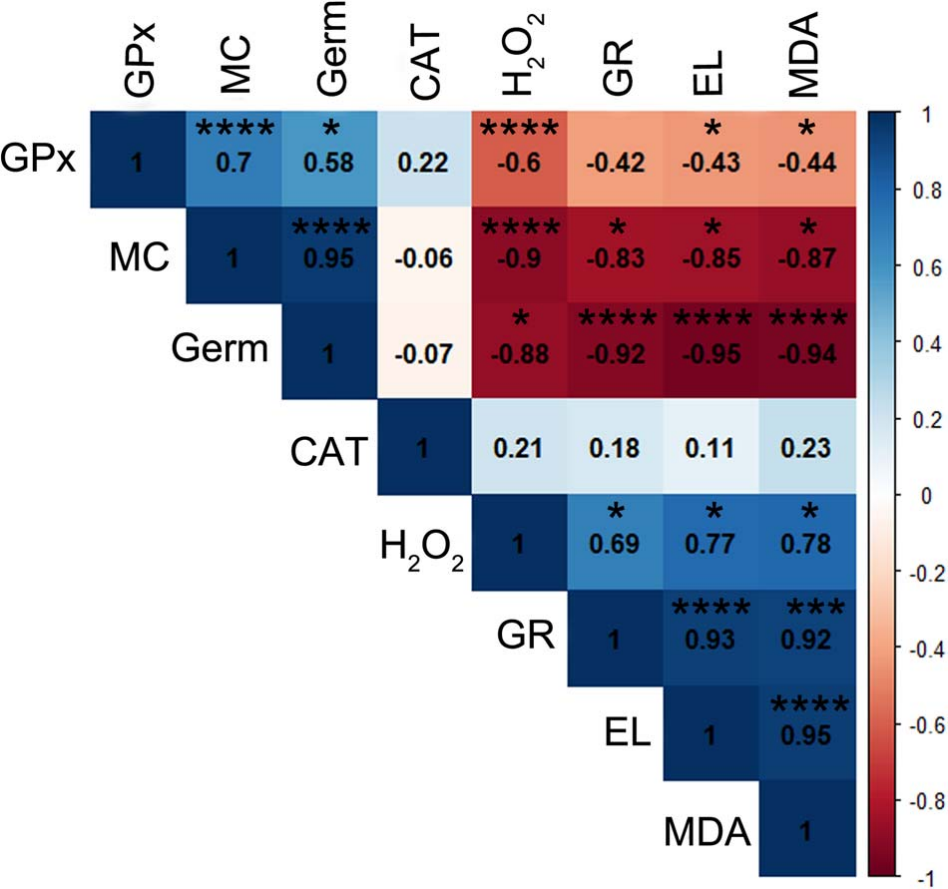
The Spearman correlation coefficient based on results from subjected to the imbibition–desiccation regime seeds between the means of activity of GPx, MC, germination (Germ), CAT activity, the level of H_2_O_2_, GR, EL and the amount of MDA. Correlation was significant at level ^*^P ≤ 0.05; ^*^^*^P ≤ 0.01; ^*^^*^^*^P ≤ 0.005; ^*^^*^^*^^*^P ≤ 0.001.

Strong negative correlations (>−0.9) between germination and GR, MDA and EL were observed after the imbibition–desiccation regime of silver maple seeds. The MC was highly positively correlated with germination and the activity of GPx and negatively (−0.9) correlated with the H_2_O_2_ level (Figure 8). In addition to a strong negative correlation with germination, GR was characterized by a very strong positive correlation with EL and the MDA content with *r* = 0.93 and 0.92, respectively. We also observed a very strong positive correlation between EL and MDA. Interestingly, the CAT activity was not correlated with any of the analyzed parameters during the drying of silver maple seeds.

**Figure 9:**
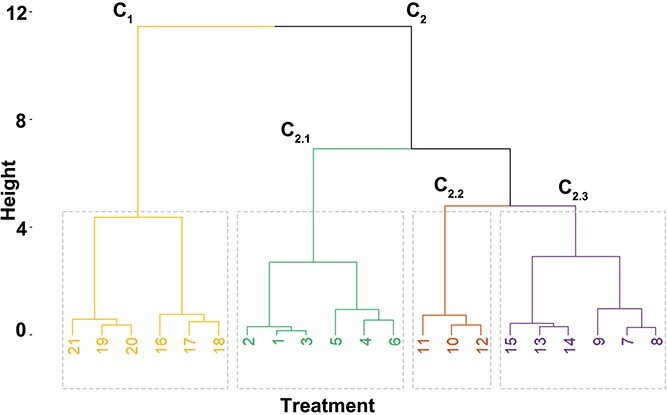
Hierarchical clustering of silver maple seeds based on the postimbibition/desiccation (*I*/*D*) activity of GPx, CAT activity, the level of H_2_O_2_, GR, EL and the amount of MDA using Ward’s method based on the Euclidean distance. The numbers in the dendrogram correspond to 1–3 control nondesiccated nonimbibed seeds at 55% MC (*C*), 4–6 seeds at 55% MC imbibed in water (*I*_W_), 7–9 seeds at 55% MC imbibed in Spd (*I*s), 10–12 seeds imbibed in water desiccated to 45% MC (*I*_W_/*D*_45_), 13–15 seeds imbibed in Spd desiccated to 45% MC (*I*_S_/*D*_45_), 16–18 seeds imbibed in water desiccated to 20% MC (*I*_W_/*D*_20_) and 19–21 seeds imbibed in water desiccated to 20% MC (*I*_S_/*D*_20_).

Hierarchical clustering analysis conducted on desiccated seeds divided the investigated seed lots into two main clusters, C1 and C2. Cluster C1 was comprised of seeds characterized by a low germinability that were imbibed in water (16–18) or Spd (19–21) and desiccated to 20% MC prior to germination. Cluster C2 could be subdivided into three subclusters C2.1, C2.2 and C2.3. Cluster C2.1 was comprised of control nontreated seeds (1–3) and nondesiccated seeds imbibed in water (4–6). Cluster C2.2 was comprised of only seeds at 45% MC imbibed in water and C2.3 included seeds imbibed in Spd at both 55 and 45% MC (Figure 9).

**Figure 10:**
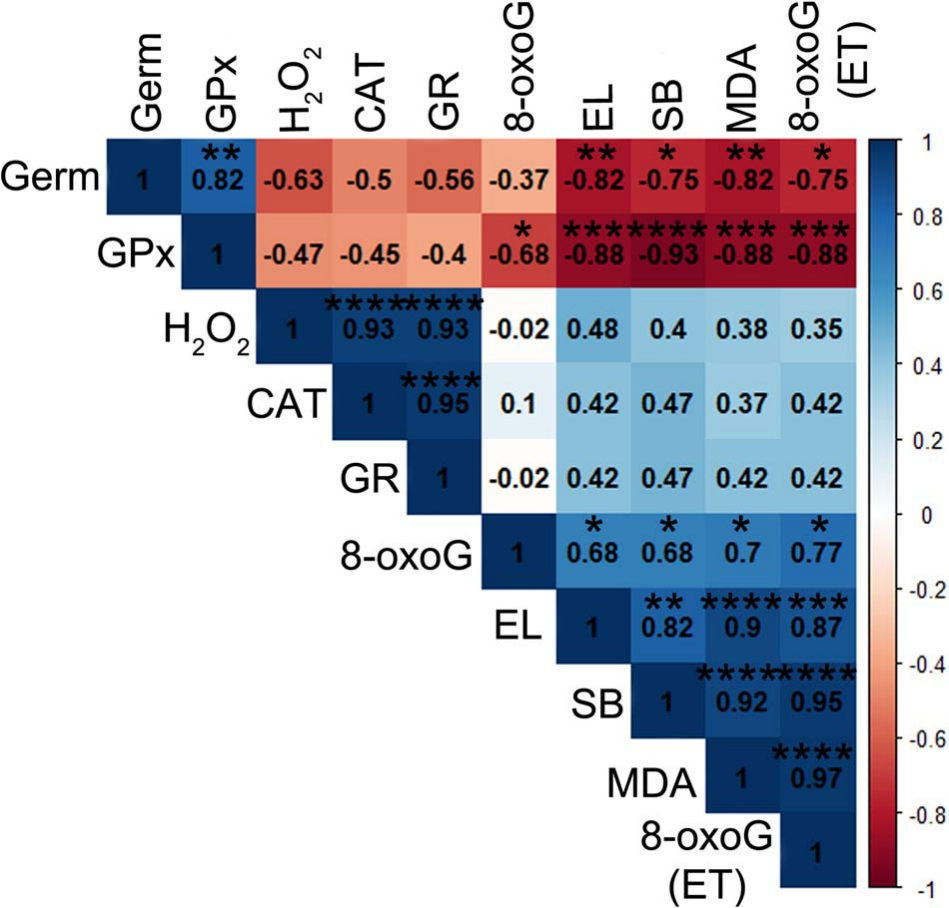
The Spearman correlation coefficient during the storage of seeds between the means of germination (Germ), the activity of GPx, the level of H_2_O_2_, CAT activity, the amount of GR, the amount of the 8-oxoG assessed by the comet assay (8-oxoG), EL, DNA SBs, the amount of MDA and the amount of the 8-oxoG assessed by the ELISA test [8-oxoG (ET)]. The correlation was significant at ^*^P ≤ 0.05; ^*^^*^P ≤ 0.01; ^*^^*^^*^P ≤ 0.005; ^*^^*^^*^^*^P ≤ 0.001.

**Figure 11:**
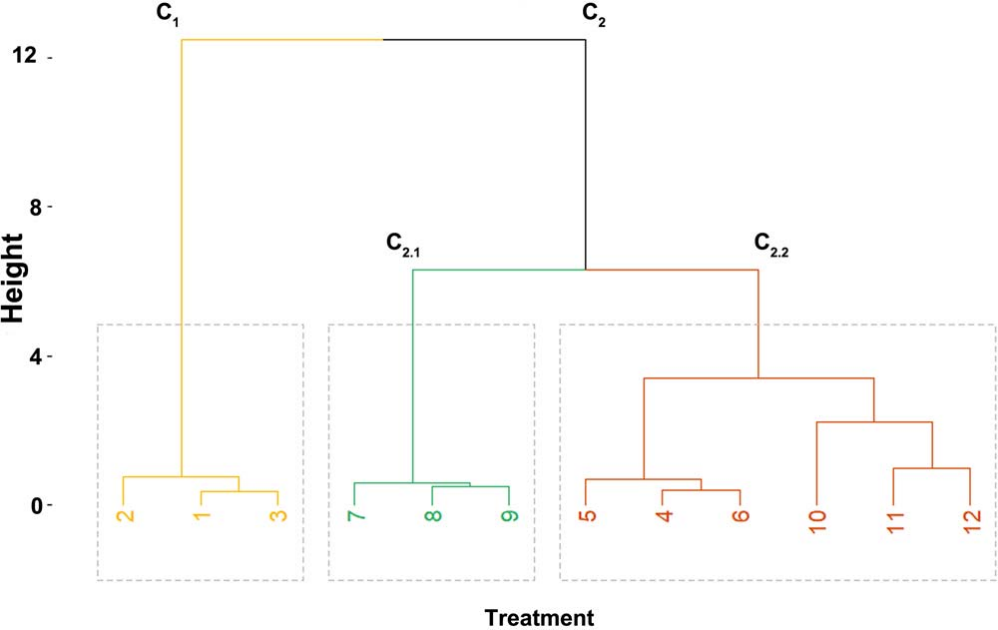
Hierarchical clustering of silver maple seeds based on the poststorage activity of GPx, CAT activity, the level of H_2_O_2_, the amount of GR, EL, the amount of MDA, the amount of 8-oxoG assessed by the comet assay, the amount of 8-oxoG assessed by the ELISA test and DNA SBs using Ward’s method based on the Euclidean distance. The numbers in the dendrogram correspond to the following: 1–3 control nondesiccated nonimbibed seeds at 53% MC (*C*), 4–6 seeds imbibed in water desiccated to 45% MC stored for 6 months (*I*_W_/*D*_45_/*S*), 7–9 seeds imbibed in Spd desiccated to 45% MC stored for 6 months (*I*_S_/*D*_45_/*S*) and 10–12 nonimbibed seeds dried to an MC of 45% and stored for 6 months (*D*_45_/*S*).

During the storage of seeds, a strong negative correlation between germination and EL, DNA SBs, the amount of MDA and the amount of 8-oxoG was observed. A strong positive correlation was observed for germination and GPx activity (Figure 10). Hierarchical clustering analysis conducted on stored seeds divided the investigated seed lots into two main clusters, C1 and C2. The first cluster consisted of nonstored control seeds, characterized by very high viability. Cluster C2 could be subdivided into two subclusters C2.1 and C2.2. Cluster C2.1 was comprised of stored seeds imbibed in Spd (*I*_S_/*D*_45_/*S*), and cluster C 2.2 comprised of both stored seeds imbibed in water and (*I*_W_/*D*_45_/*S*) stored without any pretreatment (*D*_45_/*S*) (Figure 11). During the storage of seeds, a strong negative correlation was observed between germination and EL, DNA SBs, the amount of MDA and the amount of 8-oxoG. A strong positive correlation was observed for germination and GPx activity (Figure 10). Hierarchical clustering analysis conducted on stored seeds divided the investigated seed lots into two main clusters, C1 and C2. The first cluster consisted of nonstored control seeds, characterized by very high viability. Cluster C2 could be subdivided into two subclusters C2.1 and C2.2. Cluster C2.1 was comprised of stored seeds imbibed in Spd, and cluster C2.2 was comprised of both stored seeds imbibed in water and stored seeds without any pretreatment (Figure 11).

**Figure 12:**
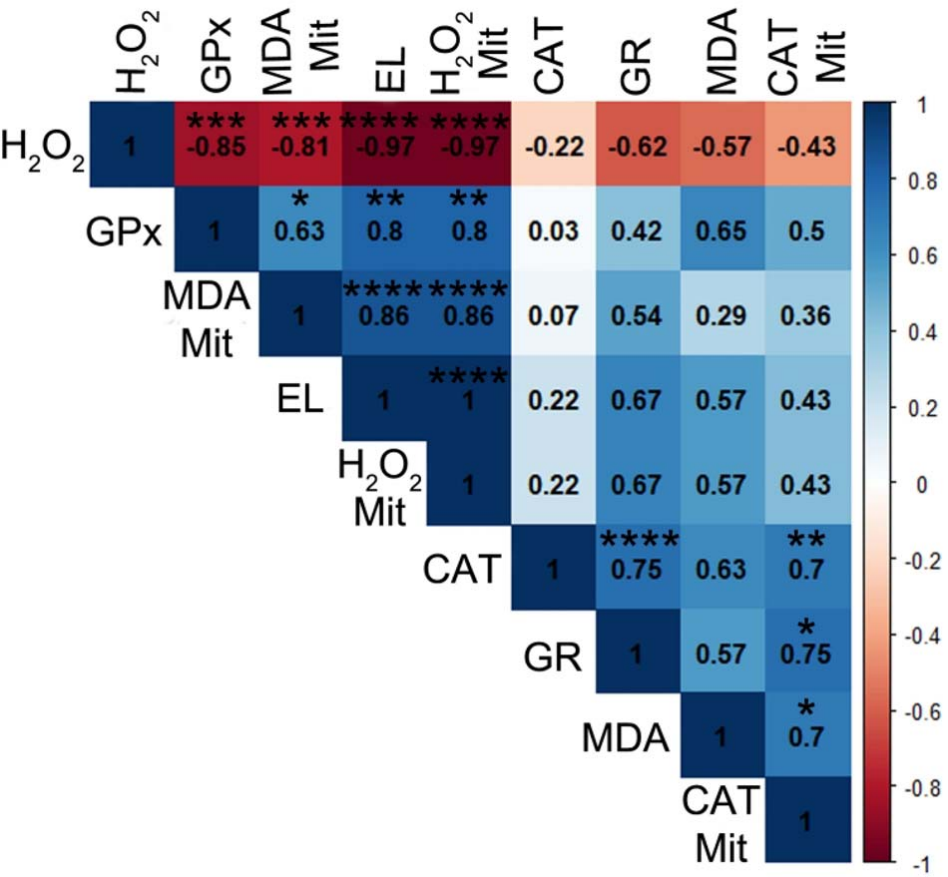
The Spearman correlation coefficient of silver maple seeds subjected to imbibition in water or Spd between the means of the level of H_2_O_2_, the activity of GPx, the amount of MDA in isolated mitochondria, EL, the level of H_2_O_2_ in isolated mitochondria, CAT activity (CAT), the activity of GR, the amount of MDA and CAT activity in isolated mitochondria (CAT Mit). Correlation was significant at ^*^P ≤ 0.05; ^*^^*^P ≤ 0.01; ^*^^*^^*^P ≤ 0.005; ^*^^*^^*^^*^P ≤ 0.001.

The level of total H_2_O_2_ was strongly negatively correlated with GPx, MDA and H_2_O_2_, as well as EL in the mitochondria of seeds imbibed in water or Spd not subjected to desiccation or storage. Mitochondrial H_2_O_2_ was positively correlated with GPx, MDA in the mitochondrial membrane and EL (Figure 12).

**Figure 13:**
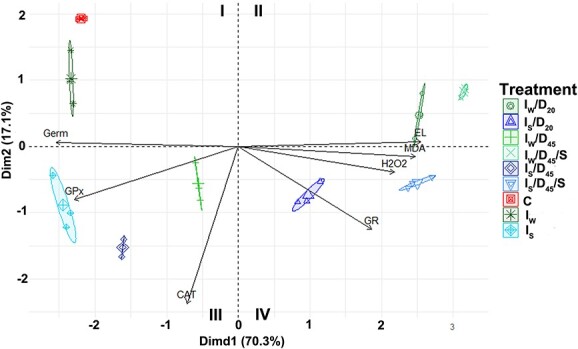
Results of PCA combining data from the aging and desiccation of silver maple seeds applied to the correlations of germination, EL, the amount of MDA, CAT activity, the activity of GPx, the level of H_2_O_2_ and the activity of GR. Biplot of principal component 1 (Dim1) and principal component 2 (Dim2).

Principal component analysis showed a high loading of EL, germination and the amount of MDA, and a moderate loading of the activity of GPx, the level of H_2_O_2_ and the activity of GR on Dim1. The activity of CAT had a major impact on Dim2, and the activity of GPx had a minor impact on Dim2. The results of PCA indicated strong negative relationships between germination and EL, the amount of MDA and the level of H_2_O_2_ (Figure 13). The first two principal components accounted for 70.3 and 17.1% of the variance, respectively (87.4%). Based on Dim1 and Dim2, the seeds of silver maple were grouped into five groups (Figure 13).

## Discussion

In order to confirm that all analyzed biochemical and structural changes result from Spd treatment and not only the imbibition process, the amount of Spd accumulated in seeds after treatment was quantified, as well as amount of other amines (Table 1). Indeed, high Spd accumulation was detected in treated seeds; nevertheless, the treatment did not change the amount of other measured amines. Consequently, in the present study, the effect of Spd on the germination of silver maple seeds was noticeable. However, in comparison with seeds imbibed in water prior to desiccation, pretreatment with Spd increased germination by 8 and 9.7% in seeds desiccated to 45 and 20% MC, respectively (Figure 2). Nevertheless, the germination capacity of seeds at an MC of 45% stored for 6 months was not different between seeds pretreated with Spd or water. It seems that in recalcitrant nondormant seeds, the desiccation stress was partially alleviated by Spd. Other researchers have indicated the positive impact of Spd on seed viability. The results obtained by Farooq et al. (2008) in dry rice seeds (*Oryza sativa* L.) indicated that several different PAs, including exogenous Spd, increased seedling viability and seed germination. Li et al. (2014) conducted research on white clover (*Trifolium repens* L.) seeds, and they observed an increase and acceleration of germination of Spd-treated seeds under stress conditions. Other authors such as Afzal et al. (2009) demonstrated in studies on tomato seeds (*Lycopersicon esculentum* L.) treated with, among others, Spd, a ~15% increase in germination. Additionally, Huang et al. (2017) confirmed an enhanced viability and germination of maize (*Zea mays* L.) seeds by ~10% under the influence of Spd. It is recognized that the positive effect of Spd on seed germination is related to the fact that PAs increase the state of cell hydration, improve amylase activity, affect the gibberellic acid content and increase H_2_O_2_ resulting from the activity of polyamine oxidase (Huang et al. 2017). However, to reiterate, in orthodox seeds that tolerate desiccation, it was observed that they can be stored for a long time in a dry state without any significant loss of viability. Here, we show that regardless of the severity of the desiccation stress, the possibility of improving the germination of recalcitrant nondormant seeds by Spd is limited up to ~10%.

Recently, Hu et al. (2020) showed that Spd enhanced the antioxidative capacity of rice seeds during accelerated aging. Indeed, Spd has been reported to be closely associated with antiaging properties (Hu et al. 2020). However, in the present study, it is clear that storage for 6 months of silver maple seeds decreased their viability. Additionally, there was no difference detected between seeds treated with water and Spd prior to desiccation and storage. Aging is manifested by the accumulation of ROS; changes in the antioxidant system; and damage to biomacromolecules, such as lipids, proteins and nucleic acids. According to the free radical theory, damage and excessive accumulation of ROS contribute to the underlying mechanisms of seed aging (Hu et al. 2020). It seems that Spd is not effective in aged seeds of low viability because it did not extend the longevity of the seeds of silver maple.

In this study, a positive effect of Spd on cell membrane integrity was observed. Both indicators of damage to cell membranes, i.e. the level of MDA (Figure 3A) and the leakage of electrolytes (Figure 3B), reached lower values in seeds treated with Spd than in seeds treated with water, except for MDA measured in seeds only after imbibition (*I*). However, after all treatments, both indicators showed higher values than in control seeds. Our results showed that Spd curbs cellular membrane damage. In contrast, Hu et al. (2020) showed that the MDA level was higher in control seeds than in seeds subjected to Spd imbibition prior to accelerated aging; however, this result was explained by the MDA increase during germination. In the current study, all parameters were measured immediately at the end of a particular regime, and Hu et al. (2020) analyzed MDA after the third day of the imbibition/germination process. The decrease in EL and MDA levels was also reported for white clover and Indian rice seeds as a result of the action of Spd (Roychoudhury et al. 2011). The reduction in membrane degradation has been recognized by the authors as an effect of increased activity of the antioxidant system resulting from Spd activity (Saha et al. 2015). However, there are several discrepancies related to ROS accumulation, oxidative damage and the activity of antioxidant system under PA treatment. For example, Huang et al. (2017) did not observe significant differences in the MDA concentration in Spd-treated maize seeds. The authors suggested that in sweet corn embryos, the accumulation of H_2_O_2_ induced by Spd does not cause oxidative damage to seed cell membranes. Moschou et al. (2008) showed that under conditions of salt stress, Spd is transported to the apoplast, where it undergoes degradation with the simultaneous secretion of H_2_O_2_. The accumulation of H_2_O_2_ in this case induced programmed cell death, while other authors suggested that H_2_O_2_ was involved in germination through a greater weakening of the endosperm cover and stimulation of the embryo to elongate the root (Huang et al. 2017). In our research, we observed an increase in the total H_2_O_2_ amount (Figure 3C) related to treatment with Spd. Significantly, the highest levels were observed in seeds with the lowest viability (*I*_s_/*D*_20_ and *I*_s_/*D*_45_/*S*). Additionally, in those seeds, CAT activity, even though improved by Spd, was the lowest. The same observation was made for GPx activity. An increase in the activity of antioxidant enzymes, including CAT, by 50% in tomato seeds treated with Spd was also reported by Afzal et al. (2009). Interestingly, in the present research, GR activity in desiccated seeds was decreased, while in aged seeds, it was improved by Spd. Glutathione reductase plays an important role in maintaining the concentration of GSH/GSSG [reduced glutathione (GSH)/glutathione disulfide (GSSG)], and as shown in the literature, the activity of this enzyme decreases in aging seeds as a result of the accumulation of ROS (Pastori and Trippi 1992, Foyer et al. 1995, Chen et al. 2013). A decrease in GR activity causes a decrease in the GSH/GSSG ratio and an increase in the GSSG level. These changes are a consequence of diminished mitochondrial integrity during aging (Kranner et al. 2006, Chen et al. 2013). Li et al. (2014) showed that in white clover seeds that were treated with Spd, a decrease in H_2_O_2_ levels in seed cells was detected, with a concomitant increase in the activity of antioxidant enzymes. Guaiacol peroxidase was particularly high in this case (Li et al. 2014). Similarly, Roychoudhury et al. (2011) observed a significant reduction in the amount of H_2_O_2_ generated in rice seeds (four times) under the influence of Spd treatment. It was suggested that exogenously administered Spd contributes to the elevation of the activity of the antioxidant system and, consequently, to reduce the concentration of ROS in seed cells. However, as in the case of membrane state indicators, all previous studies were performed on orthodox seeds that have efficient antioxidant systems. Here, in seeds of low viability, the limited activity of antioxidant enzymes (CAT and GPx) no longer balanced H_2_O_2_ resulting either from possible polyamine oxidase activity or from severe desiccation- and aging-related processes. Additionally, PCA showed that the incubation of seeds in Spd had the most significant effect on the activity of CAT and, to a lesser extent, on the activities of GR and GPx (Figure 13), although this effect did not improve the germination capacity by more than 10%.

Reactive oxygen species have a high potential to induce DNA SBs that can be extremely deleterious and threaten genomic integrity (Plitta-Michalak et al. 2022*a*). Therefore, as the level of H_2_O_2_ was the highest and germination capacity the lowest in seeds subjected to Spd treatment followed by mild desiccation and storage (*I*_s_/*D*_45_/*S*), our interest was directed to the possible accumulation of DNA oxidative damage during storage. Statistical analyses showed a negative correlation between the presence of DNA damages and germinability (Figure 10). In seeds desiccated to an MC of 45% and stored without any pretreatment, a high level of 8-oxoG was detected in both assays (Figure 4B and C). However, it was also significant that treatment with Spd resulted in the most substantial decrease in the level of oxidized G, which may have resulted from either the activation of the repair system that removes oxidative damage or the antioxidative properties of Spd and protection from ROS activity toward DNA. Importantly, the amounts of 8-oxoG and SBs, although the lowest in Spd-treated seeds, were higher than those in control nontreated seeds. The observed high number of DNA SBs in all stored seed lots may result from not only DNA damage but also DNA base excision repair as SBs are present as a result of damaged base excision and the subsequent removal of the residual DNA backbone (Figure 4A). Therefore, a high percentage of tail DNA may be a consequence of ongoing DNA repair, including incomplete DNA SB repair as well as coexisting ROS-related damage to DNA at any time point. The observed state of DNA integrity is the consequence of these two opposite processes. However, even though Spd alleviated oxidative damage to DNA, and this phenomenon was also observed by others for orthodox seeds (Taie et al. 2019) and other models (Khan et al. 1992, Oh and Kim 1998, Kim et al. 2006), it is clear that in aged nondormant recalcitrant seeds, protection imposed by Spd toward oxidative attack on DNA did not rescue seed viability.

Finally, the effect of Spd on mitochondria, which are one of the main sites of ROS generation, and an important energy center of the cell, was investigated (Yin et al. 2016, Ratajczak et al. 2019*b*). In our study, we observed lower levels of both MDA and H_2_O_2_ in Spd-treated mitochondria along with an increase in CAT activity. Similar results were obtained by Pan et al. (2016) in the mitochondria of two tomato varieties, in which, under conditions of salinity stress and alkalinity, exogenous Spd can effectively mitigate mitochondrial damage by reducing H_2_O_2_ and MDA levels and mitochondrial membrane permeability. Moreover, differences in the structure of mitochondria from seeds treated with Spd were observed as they were elongated with numerous cristae that have a crucial role in metabolic activity. There is a correlation between the modulation of the shape and network of mitochondria and the energy state of the cell. Oxidative stress causes mitochondria to lengthen, protecting mitochondria from degradation and promoting mitochondrial ATP production (Cogliati et al. 2016).

Additionally, the impact of Spd on mitochondria was also measured as a function of the OCR. To the best of our knowledge, the influence of exogenous Spd on respiration processes in seeds has not yet been studied. Some attempts have been made on the leaves of *Arabidopsis thaliana* L. and *Troyer citrange*, where exogenous Spd increased the respiration rate (Anjum 2009, Andronis et al. 2014); however, this effect is not desirable in the case of the long-term storage of seeds. The measurements of the OCR in seed embryonic axes treated with exogenous Spd revealed that this compound stabilized the respiration rate in comparison with control embryonic axes imbibed in water. This observation is consistent with the Spd-related reduction in the mitochondrial oxidative milieu (decrease in H_2_O_2_ and increase in CAT activity) and the maintenance of mitochondrial membrane stability. Moreover, the impact on mitochondria and a highly desirable positive effect in reducing the intensity of seed metabolism may contribute to a slight increase of 8–10% in the germination capacity in desiccated seeds. In our studies, we observed the effects of Spd at both cellular and subcellular levels. Although Spd affects the cellular redox environment, protects DNA from damage to some extent and improves mitochondrial activity as well as the activity of mitochondrial antioxidative enzymes, this compound does not remove all of the negative effects of ROS accumulation. However, Spd clearly affects multiple cellular processes. This effect is particularly visible in hierarchical cluster analyses (Figure 9), where seeds subjected to imbibition in water or Spd and desiccation up to 45% MC were separately grouped into clusters C2.2 and C2.3. Interestingly, based on measured biochemical and structural parameters, seeds only imbibed in Spd and subjected to imbibition and desiccation showed the highest similarity. Also, in the case of seeds desiccated to 20% of MC (C1), the seeds were grouped into separate clusters base on imbibition difference. The effect of Spd is visible in stored seeds as seeds treated with Spd (C2.1) are grouped in a separate cluster based on measured parameters (Figure 11). The PCA analysis (Figure 13) also showed that Spd improved the germinability of treated seeds as there is a shift toward quarters I and IV detected for seeds imbibed in Spd in comparison with seeds imbibed in water. Moreover, seeds treated with Spd are grouped in quarters III and IV, suggesting Spd impact on CAT; however, in silver maple seeds, CAT activity is not correlated with germinability (Figure 10). Therefore, further research on the impact of Spd on seeds seems to be beneficial for a better understanding of redox regulation in seeds and expanding their storage feasibility.

## Data Availability

Experimental data and materials are available to third party academic researchers upon reasonable request.
